# Prof. Mott, Quacks and Quack Nostrums

**Published:** 1854-04

**Authors:** 


### EDITORIAL DEPARTMENT.

---

### *Prof Mott, Quacks and Quack Nostrums.*

We confess to no little surprise and astonishment when we have, from time to time, observed the honored name of this distinguished man attached to certificates endorsing the qualifications and capacity of some itinerant charlatan or prating quack, we were well assured that he despised all such and that he lamented the existence of so much ignorance and credulty, constituting their capital and their very life blood, we felt assured that he would revolt at proposition to affix his signature to any paper, recommending to public favor, any of the vile nostrums now flooding the country and yet, to several of these we have seen *his* name, together with other prominent members of the profession placed there by the knaves who make and vend them in order more readily to impose upon the credalety and weakned of a certain class of people.

The letter below "defines his position," and is an emphatic caveat against the further use of his name. The members of the profession will rejoice to see this because, like ourselves, they were astounded that he could lend his name to so base a purpose and if not so, which was most likely, then all were shocked that any individuals could in open day perpetrate so foul a forgery.

Whilst upon this subject we would venture the enquiry whether a similar outrage has not been committed in the use of the names of respectable ministers of the gospel, whose names too find affixed regularly *signed, sealed* and *delivered* to these instruments fulminating these cheats into conspicuity and public favor.

We could not for a moment beleive that a learned body of devout men, wearing the sacred robes of their divine appointment, would give countenance to humbugs and base cheats. Rather were we taught to expect that it would constitute a part of their sacred and solemn duty to warn the people against their use, as dangerous to life, and inimical to health. The very secresy in which the constituants of these compounds are enveloped would be sufficient ground of suspicion, and of instant and unqualified rejection, such empiricism is an extensive system of deception, and as such they are bound by the most sacred obligations, which they are to themselves community and their vows, to oppose and condemn. The advocates and promulgators of *truth*, they should hate every form of deception, dishonesty, and fraud, and therefore they cannot, consistently with their sacred obligations favor hypocracy in any form whether it leads to moral or physical evil. The avowed enemies of all heresy, schisms and *isms*, and every false system caculated to lead the mind estray from the path of truth we would hope to find them enroled on the side of those time-honored institutions whose capacities for good have been severely tested by a long experience. We do not believe therefore that the clergy, as a body would blindly endorse a remedy however much vaunted without knowing its constituant properties but we must confess we are much staggered when we see a few persist in it even after its analysis has been made and its dangerous properties have been declared.

Although our clergy are the advocates for the highest qualifications and attainments, yet in some instances are found a few giving "aid and comfort" to certain systems of empiricism. We are glad to know that among the truly learned of the "black cloth" these instances are rare. On the contrary, we have heard many in the sacred desk, expose the knavery of the makers and venders of patent nostrums, and the dangers of the use of these vile compounds; and we have now in our mind's eye, those who have felt themselves bound to warn the people against all clap-trap heresies in medicine, itinerant-montebank lecturers, and *quasi doctors.* We honor such for their honesty and the high estimate they place upon what is a solemn and a sacred duty.

What confidence can an enlightened community place in the opinions and judgment of any man who follows every *will-o-the-wisp* intended to beguile and lead its credulous followers into darkness, and then in turn to fade into oblivion? Towards such there is felt a pity for their blindness and weakness, and their opinions are held in distrust by all sensible men. And yet they rail against all *isms* and *schisms* in religion, and in holy amazement they anathematize the mad followers. And yet it would not be more absurd in us to follow the faith of the "Millerites" or the "Mormons," than for them to be led away, the willing subjects and dupes of systems equally ridiculous, unreasonable and absurd.

But scientific men in general are not prone to follow these phosphorescent luminaries and false lights. They are engaged continually in their inquiries after truth and facts through a process of inductive reasoning, and they are taught to detest quackery too much to follow after the trail of every theological comet of a fortnights duration. They are too well aware of the aching desire by the credulous for something wonderful and novel, and they too well know how successfully charlatans can impose upon these, to countenance canting and hypocracy.

As a body, medical men pursue a course of non-interference with the sacred duties of the clergy, nor are they active propagandists in favor of any particular creed or religious tenets, but leave it to those whose sacred duty it is to remedy the moral ills of their fellow man. We ourselves claim instructiion from them, accrediting their right to know more of these matters. But while we extend this courtesy to *them*, they should "do unto others as others do unto them," and not enter the field with their weighty influence in the advocacy of humbugs and clap-traps. It betrays an ignorance upon medical subjects, a weakness, a desire for officious meddling beneath the dignity of a sound Divine, to say nothing of the reckless disregard for health and life. The profession of medicine can take care of itself, and it *will* expose the conduct of those who will step out of their own legitimate sphere and obtrude themselves upon their province. We never have nor will we ever oppose the slightest barrier to the discharge of their duties. We have never, by the slightest intimation, sought to lessen the confidence of the community in their sincerity or piety: on the contrary we have ever aimed to hold up their hands, and sustain the true orthodox minister of whatever faith, even when doubts have been expressed in regard to their capacity or faithfulness in their stewardship. We shall be regulated by the principle of *meum et tuum*, and would commend the adoption of it by others. We have said more on this subject than we intended at this time, but will recur to it again.

But now to the disclaimer of Dr. Mott.

New York, Dec. 26, 1853.

Ed. Ohio Med. and Surg. Journal,

Sir:—Will you be so kind as to correct a mis-statement in the Nov. number of the *Ohio Medical and Surgical Journal*, of which you are Editor.

I never recommended Dr. Hartly as an Occulist or Curist. If he refers to me, therefore, it is wholly unauthorized.

In various directions of our country, I find myself set forth in connection with, Pills, Powders and Balsams, which I know as much of as I do of Dr. Hartly as an Occulist.

I hope you will give me the pleasure of seeing your Journal more frequently hereafter. Respectfully yours,

VALENTINE MOTT.

In our next we will publish a list of "certified witnesses" compiled from the advertising mediums of various quack nostrums. Let the names be published in the different Journals near their several localities, that they may be favored with an opportunity of imitating the example of Dr. Mott. If not give them their due rank in the profession, "being joined to their idols, let them alone."

